# Unexpected development of tongue squamous cell carcinoma after sclerotherapy for the venous malformation: a unique case report and literature review

**DOI:** 10.1186/1746-1596-8-182

**Published:** 2013-11-04

**Authors:** Gang Chen, Xu Cai, Jian-Gang Ren, Jun Jia, Yi-Fang Zhao

**Affiliations:** 1 State Key Laboratory Breeding Base of Basic Science of Stomatology (Hubei-MOST) and Key Laboratory of Oral Biomedicine Ministry of Education, Wuhan University, Wuhan, China; 2 Department of Oral and Maxillofacial Surgery, School and Hospital of Stomatology, Wuhan University, Wuhan, China

**Keywords:** Venous malformation, Sclerotherapy, Squamous cell carcinoma

## Abstract

**Background:**

Sclerotherapy is a common and effective treatment for venous diseases, including venous malformations (VMs), which are common vascular anomalies in the oral and maxillofacial regions. However, the safety of sclerotherapy has not been fully elucidated. Occasionally, patients who underwent sclerotherapy may present diverse but minor side effects such as erythema, swelling, pain, tenderness, hyperpigmentation, skin ulceration and necrosis.

**Case presentation:**

Here we report a unique case of a 65-year-old female patient presented with an original VM lesion on the right side of the tongue. Intralesional sclerotherapy and followed surgical resection resulted in major remission of the original lesion, without recurrence during a 3-year follow-up. However, two years later, the patient was again referred to us for a painful mass on the right side of the tongue that gradually enlarged for 1 month. The mass was biopsied under local anesthesia after complete systematic examination, and the result indicated a well-differentiated squamous cell carcinoma (SCC). Then, the patient underwent right neck dissection, extensive resection of the SCC, reconstruction of the defect with forearm flap, microvascular anastomosis, and repair of the forearm defect with free abdomen skin graft.

**Conclusion:**

To the best of our knowledge, this is the first study to document the development of oral SCC after sclerotherapy for VM, underscoring the need for long-term follow-up.

**Virtual slides:**

The virtual slides for this article can be found here: http://www.diagnosticpathology.diagnomx.eu/vs/1897394831087742.

## Background

Venous malformations (VMs), formerly known as “cavernous hemangiomas”, are the most common slow-flow vascular malformations that have a propensity to form in the oral maxillofacial regions. VMs have an estimated incidence of 1 to 2 in 10,000 births and a prevalence of 1% [1]. Unlike infantile hemangiomas, VMs never regress and most of them are sporadic and unifocal. They have no sex preponderance, but they have an age-dependent variation in penetrance, which peaks at approximately 20 years old [2]. Clinically, VMs located in the oral region often cause serious problems in speech and swallowing, and they may even be life threatening because of bleeding, expansion, or obstruction of vital structures. Given the anatomic and histologic characteristics of the oral and facial regions, sclerotherapy is the preferable treatment option to reduce the volume of the lesions. However, the safety of sclerotherapy has not been fully elucidated. This case report describes a 65-year-old female patient who suffered squamous cell carcinoma (SCC) of the tongue after sclerotherapy for VM.

## Case presentation

**Figure 1:**
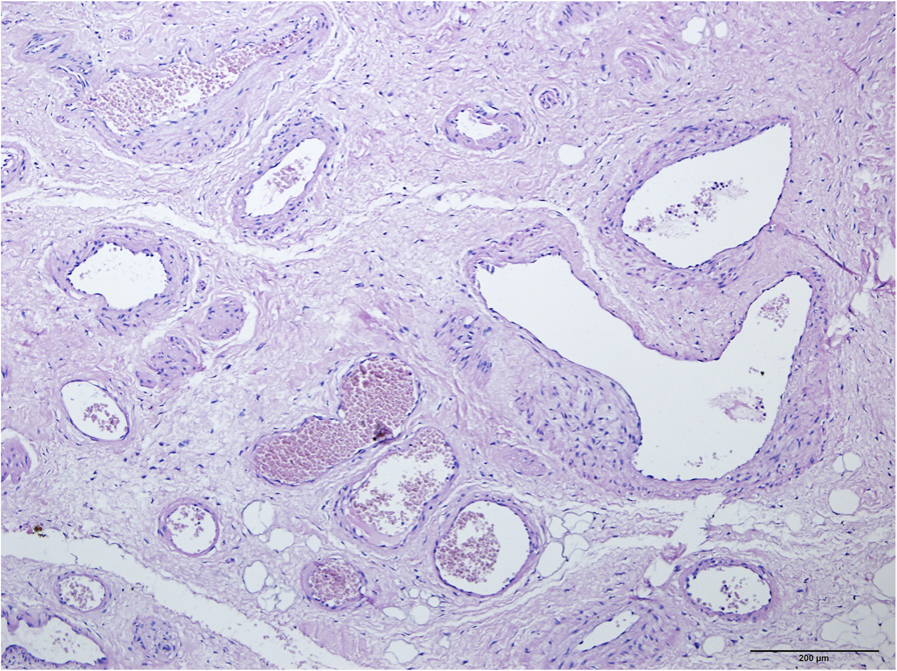
**Biopsy result of the original VM lesion.** Hematoxylin and eosin staining of original VM lesion showed irregular venous-type channels, which were varied in size but surrounded by thickened lumen walls.

In January 2007, a 65-year-old female patient was admitted to our department with chief complaint of a soft mass in the tongue. The mass was initially noticed 35 years ago and gradually enlarged with age. Physical examination showed a port-wine stained mass measuring 5 cm × 5 cm on the right side of the tongue. The mass was soft to the touch, compressible, and nontender. No ulceration of the oral mucosa was observed, and no cervical lymph node was palpable. Considering the aforementioned clinical features, the patient was presumptively diagnosed with VM of the tongue. After that, the subsequent sclerotherapy plan was designed. Briefly, pingyangmycin (8 mg) was initially injected into the lesion under local anesthesia, followed by laser therapy (Nd:YAG laser) 2 months later with the purpose to enhance the treatment effect for superficial lesions. Then, the intralesional pingyangmycin injection (8 mg) was repeated two times alternating with a single sodium morrhuate injection (150 mg in 3 ml). The mass gradually hardened and decreased in size, and was surgically resected for the major part after 6 months. The pathological examination result of the removed lesion showed a typical manifestation of VM after sclerotherapy (Figure 1), as demonstrated by irregular venous-type channels, which were varied in size but surrounded by thickened lumen walls. The patient was satisfied with the outcome, with no recurrence during the 3-year routine follow-up.

**Figure 2:**
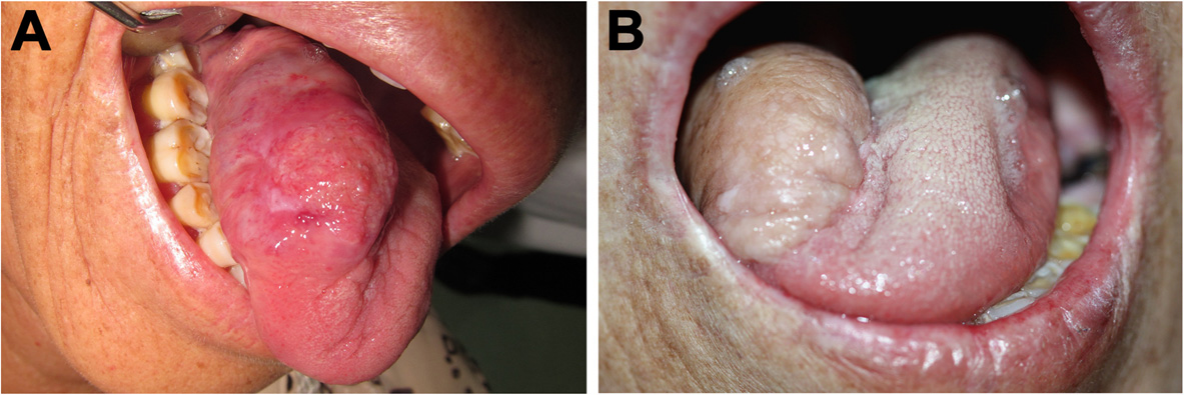
**Clinical appearance. (A)** Preoperative view of tumor showing a solid lesion with a cauliflower-like surface on the right side of the tongue. **(B)** Postoperative view of tongue at 6 months after hemiglossectomy and reconstruction with vascularized forearm flap.

The patient was again referred to our department for a painful mass in the tongue that gradually enlarged for 1 month in 2012. The patient had no history of alcohol abuse and smoking, and had no family history for malignant tumors either. After oral and facial examination, we found a 2.5 cm diameter poorly demarcated solid lesion with a cauliflower-like surface on the right side of the tongue (Figure 2A). No cervical, submandibular, and submental lymphadenectasis was observed on both sides. The mass was biopsied under local anesthesia after complete systematic examination, and the result indicated a well-differentiated SCC. Then, the patient underwent right neck dissection, extensive resection of the SCC, reconstruction of the defect with forearm flap, microvascular anastomosis, and repair of the forearm defect with free abdomen skin graft (Figure 2B). No evidence of recurrence or metastasis was found during routine follow-up.

### Pathological and immunohistochemical findings

**Figure 3:**
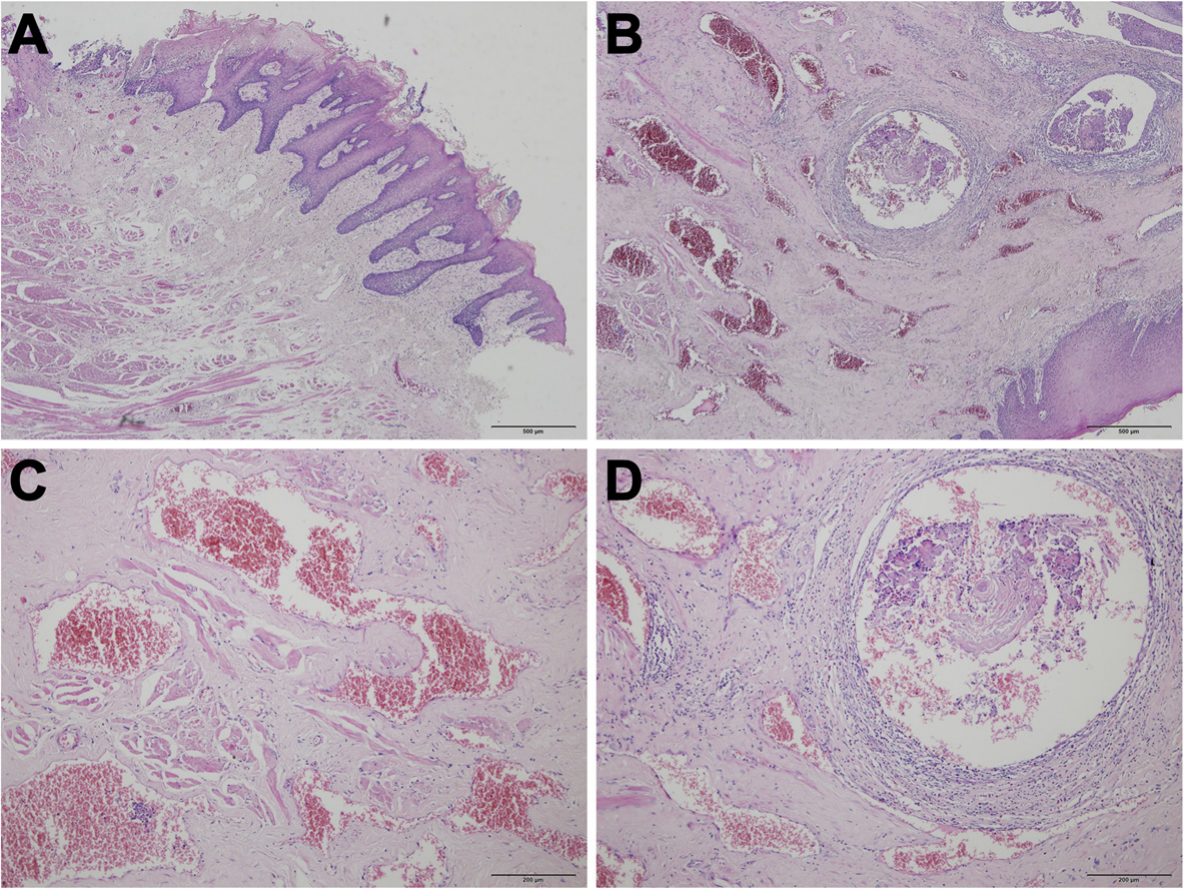
**Histopathologic features of the surgical SCC lesion. (A)** Hematoxylin and eosin staining of the lesion showing a well-differentiated squamous cell carcinoma. **(B)** Peripheral region showing residual venous malformations (**C**, magnification) and phlebolith (**D**, magnification).

**Figure 4:**
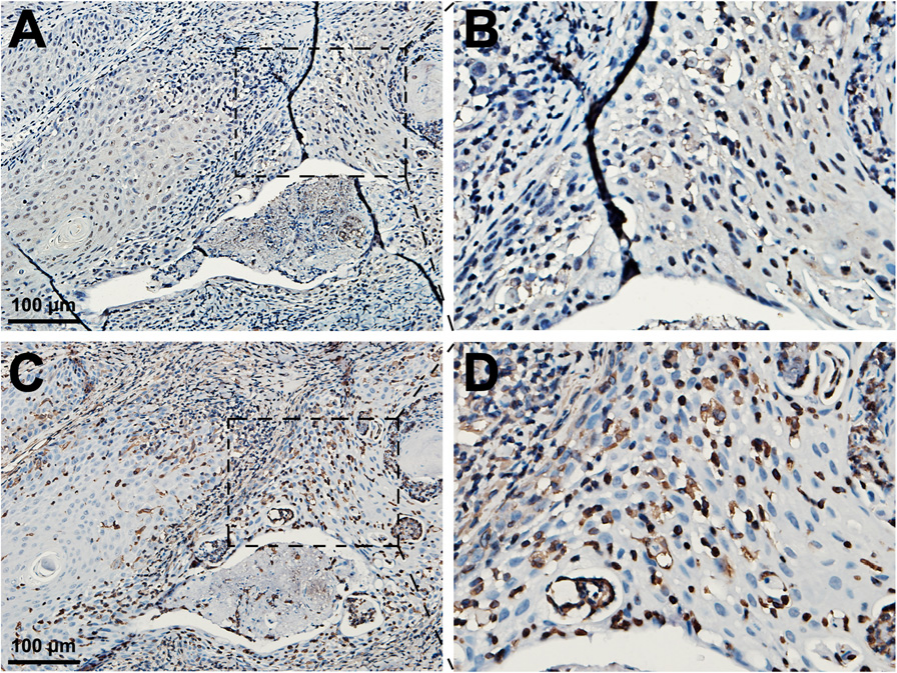
**Immunohistochemical staining for mesenchymal markers in the surgical SCC lesion after sclerotherapy. (A)** N-Cadherin staining was diffusively positive in the lesion (**B**, magnification). **(C)** Vimentin staining was strongly positive in the lesion (**D**, magnification).

**Figure 5:**
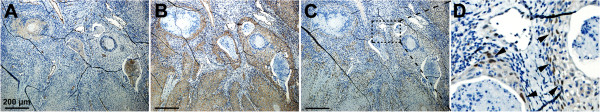
**Immunohistochemical staining for EndoMT-related markers in the surgical SCC lesion after sclerotherapy. (A)** The continuous expression of CD34 (endothelial cells) of the vessels within residual VMs was obviously damaged. **(B)** Strong expression of α-SMA (smooth muscle cells) around the vessels within residual VMs, or even invaded across the endothelial layers to fill the venous lumen. **(C)** Positive nuclear location of slug in the inner layer of the vessels within residual VMs (**D**, magnification).

The post-operation pathological examination not only confirmed the diagnosis of well-differentiated SCC with keratin pearls, but also revealed the surrounded residual VMs and phlebolith (Figure 3), which was probably due to the incomplete resection of original VM lesion, considering its benign nature as well as the normal appearance and function of tongue. Moreover, a large amount of mesenchymal-like cells, leading to formation of small tubular structures, were observed at the boundary area between SCC and residual VMs. In order to gain more insights into the possible relationship among original VM lesion, sclerotherapy and SCC development, we then performed the immunohistochemical analysis for several cell markers. Firstly, we found that N-Cadherin and vimentin, two well known mesenchymal cell markers, were strongly expressed in the present case, especially within the juncture between new-developed SCC and residual VMs (Figure 4). More importantly, during our attempt to understand the possible mechanisms underlying the increased mesenchymal components, we unexpectedly found that the expression of CD34 (endothelial cells) within residual VMs was damaged (Figure 5A), and replaced by increased expression of α-SMA (smooth muscle cells) around the vessels and even invaded across the endothelial layers to fill the venous lumen (Figure 5B). In addition, the nuclear location of slug could be frequently observed in the inner layer of the vessels (Figure 5C), suggesting the occurrence of an EndoMT-like process after sclerotherapy. Meanwhile, we also revealed that α-SMA was also strongly expressed in the boundary area between new-developed SCC and residual VMs, as well as the invasive front of SCC (Figure 5B).

**Figure 6:**
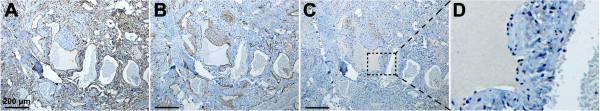
**Immunohistochemical staining for EndoMT-related markers in the surgical VMs without sclerotherapy. (A)** The expression of CD34 at the intima of the vessels was almost continuous. **(B)** The perivascular α-SMA psotive cells were focally scant or even absent. **(C)** Positive nuclear location of slug was nearly undetectable **(D**, magnification**)**.

For more evidences, several resected VM specimens without other treatment history were retrieved from the Department of Oral Pathology, Stomatology School of Wuhan University, and the activation status of EndoMT was evaluated by detecting the expression levels of CD34, α-SMA and slug. The representative immunohistochemical results were shown in Figure 6. Compared with pingyangmycin-treated VMs, the venous channels within untreated VMs were varied in size and covered by very thin lumen walls. Moreover, in most of untreated VM samples, the expression of CD34 at the intima of the vessels was demonstrated to be continuous (Figure 6A), and the perivascular α-SMA^+^ cells were focally scant or even absent (Figure 6B). In addition, the lumens of most vessels in untreated VMs contained blood, and the rest may be empty or contain only sedimented proteins or erythrocytes. Most importantly, positive nuclear location of Slug was nearly undetectable in these untreated VM samples (Figure 6C). All these above findings suggested that the process of EndoMT was in an inactivated status in untreated VMs, and sclerotherapy with pingyangmycin might thereby increase the mesenchymal components through activation of the EndoMT process.

## Discussion

Sclerotherapy is a common treatment method for venous diseases. In most cases, the treatment is effective, with a low frequency of complications. Previous studies have shown that the complications of sclerotherapy could be classified as local or systemic. Local complications include any transient adverse effects, such as peripheral nerve palsy, skin blistering, tissue fibrosis, or skin necrosis. Systemic complications include death and permanent adverse effects, such as deep venous thrombosis, pulmonary embolism, and other cardiovascular or pulmonary events [3].

In the present study, we report a case of 65-year-old female patient who suffered SCC of the tongue after sclerotherapy for VM. To our best knowledge, this is the first study to document the development of oral SCC after sclerotherapy. Nevertheless, several sporadic cases of esophageal cancer development following sclerotherapy have been reported for esophageal varices [4-9], and most of the carcinomas are SCC. Moreover, in 2012, Hashimoto et al. also reported a unique case of papillary renal cell carcinoma after sclerotherapy for simple renal cyst [10]. However, the cause-effect relationship between sclerotherapy and carcinogenesis is still poorly understood, especially concerning whether sclerotherapy per se is linked to carcinogenesis or just the sclerosants, because the commonly used sclerosants were in various kinds, including absolute ethanol, pingyangmycin, sodium morrhuate, bleomycin, polidocanol, sodium tetradecyl sulfate (STS). Among those reported cases of carcinoma development after sclerotherapy, ethanolamine oleate was used in three cases, polidocanol was used in two cases, and the rest used sodium morrhuate, sotradecol, minocycline, and ethanol (Table 1).

**Table 1:** Information of patients who suffered carcinoma development after sclerotherapy

**Case**	**Patient age(years)/sex**	**Lesion location**	**Primary lesion**	**Duration of carcinoma development (months)**	**Sclerosants used**	**Total dose**	**Reference**
1	54.7 ± 6/M	Esophagus	Esophageal varices	12-30	Polidocanol	90-310 ml	[3]
2	Unknown	Esophagus	Esophageal varices	24	Sodium morrhuate	10 ml	[4]
3	Unknown	Esophagus	Esophageal varices	21	Ethanolamine oleate	45.5 ml	[4]
4	52/M	Esophagus	Esophageal varices	36	Polidocanol	117 ml	[5]
5	58.3 ± 5.0/Unknown	Esophagus	Esophageal varices	9-33	Ethanolamine oleate	51.0 ± 18.9 ml	[6]
6	56/M	Esophagus	Esophageal varices	54	Ethanolamine oleate	70 ml	[7]
7	Unknown	Esophagus	Esophageal varices	5-48	Sotradecol	30 ml	[8]
8	47/F	Kidney	Simple renal cyst	36	Minocycline and Ethanol	200 mg	[9]
9	65/F	Tongue	Venous malformation	65	Pingyangmycin and Sodium morrhuate	174 mg	Present case

Pingyangmycin and sodium morrhuate were the sclerosants used in this case. Pingyangmycin is a bleomycin A5 derivative. Given its rare side effects, low cost, and easy availability, it is widely used in China for over 20 years. Zheng et al. used pingyangmycin to treat VMs in the oral and maxillofacial regions, and showed that 134 of 179 patients (74.9%) were improved or cured [11]. Pulmonary fibrosis is the most serious complication of pingyangmycin, but the risk is minimal when the dose per treatment is less than 1 mg/kg per session, with a total dose of 5 mg/kg. Our previous study also found that alternating injections of pingyangmycin and sodium morrhuate is more effective for VMs than using them alone [12]. However, the safety of this therapeutic modality has not been fully elucidated yet. Here, we report a 65-year-old female patient who suffered SCC of the tongue after sclerotherapy with pingyangmycin and sodium morrhuate. This case is unique because it presents an oral mucosal carcinoma developed from a VM after sclerotherapy, and no ulceration occurred before the carcinoma development. Also, this is the first report to link the sclerosant pingyangmycin with carcinogenesis. Nevertheless, there are still several puzzles to be solved for their potential relationship.

In that regard, the post-operation pathological examination result have showed that the well-differenced SCC was surrounded by residual VMs and phlebolith, and meanwhile revealed that a large amount of mesenchymal-like cells were located in the boundary area between SCC and residual VMs. These observations suggested a potential relationship between SCC development and sclerotherapy, and also promoted us to hypothesize that whether the carcinoma development after sclerotherapy was associated with the increased mesenchymal components, because a plenty of previous studies have indicated the promotive roles of stromal microenvironment during carcinogenesis [13].

Based on the above concerns, we then performed the immunohistochemical analysis for several mesenchymal markers. Firstly, we found that N-Cadherin and vimentin were strongly expressed in almost the entire specimen, including the invasive front of SCC, the boundary area between SCC and residual VMs, as well as the perivascular walls of VMs, which suggested the significantly increased mesenchymal components. Then, by comparison with those surgical VMs without sclerotherapy, we confirmed the increased expression of α-SMA around or invaded across the vessels of residual VMs in the present case. Importantly, nuclear location of slug, a key member of the EMT-related transcription factors, was also frequently found in the inner layer of the vessels, while positive nuclear location of slug was nearly undetectable in the VM samples without sclerotherapy. All these results indicated an occurrence of EndoMT-like process in this case after sclerotherapy with pingyangmycin. Notably, previous studies also indicated the truth that sclerotherapy could significantly increase the mesenchymal components (mainly fibroblasts) through an EndoMT-like process [14,15]. More specifically, our very recent study revealed that sclerotherapy-induced EndoMT might be associated with the activation of mTOR pathway [15]. Taken all the above findings into consideration, we suspected that EndoMT might be an important mechanism contributing to the sclerotherapy of venous diseases, but might be also a possible link between sclerotherapy and carcinoma development. Besides, we speculated that sclerotherapy could lead the lesions and adjacent tissues to become harden and constrict, so that the mechanical force and heat stimulation may also play a potential role during the process of carcinogenesis. Also, it has been widely accepted that carcinoma development involves the progressive accumulation of genetic abnormalities as well as the alteration of local environment [16-18]. Thus, further study will be required to determine whether sclerotherapy could lead to genetic abnormalities or environmental alteration.

## Conclusion

We herein described an unusual case of 65-year-old female patient who suffered SCC of the tongue after sclerotherapy for VM, and meanwhile provided some underlying clues for the possible link between sclerotherapy and carcinoma development. Therefore, we emphasize that long-term routine follow-up after sclerotherapy is necessary, and the side effects and complications of sclerosants should not be overlooked, especially any neoplasm in oral and facial regions.

## Consent

Written informed consent was obtained from the patient for publication of this case report and any accompanying images. A copy of the written consent is available for review by the Editor-in-Chief of this journal.

## Abbreviations

VM: Venous malformation; SCC: Squamous cell carcinoma; α-SMA: Alpha-smooth muscle actin; STS: Sodium tetradecyl sulfate; EndoMT: Endothelial-to-mesenchymal transition.

## Competing interests

The author declares to have no competing interests.

## Authors’ contributions

GC and XC performed the literature review and drafted the manuscript. JGR carried out the tissue staining. JJ and YFZ treated this patient clinically, and made contributions for the acquisition of clinical data. All authors read and approved the final manuscript.

## References

[B1] Eifert S Villavicencio JL Kao TC Taute BM Rich NM Prevalence of deep venous anomalies in congenital vascular malformations of venous predominance J Vasc Surg 2000 8 462 471 10709058

[B2] Brouillard P Vikkula M Genetic causes of vascular malformations Hum Mol Genet 2007 8 Spec No. 2:R140-9 10.1093/hmg/ddm211 17670762

[B3] Guillemot F Bonnière P Bretagne JF Ancelin JP Raoul JL Plane C Cortot A Paris JC Esophageal cancer and endoscopic sclerosis of esophageal varices: a fortuitous association [abstract]? Gastroenterol Clin Biol 1988 8 858 861 3220235

[B4] Kokudo N Sanjo K Umekita N Harihara Y Tada Y Idezuki Y Squamous cell carcinoma after endoscopic injection sclerotherapy for esophageal varices [abstract] Am J Gastroenterol 1990 8 861 864 2196786

[B5] Macias Rodriguez MA de la Cruz MJ S Iglesias Arrabal M Martin Herrera L Esophageal carcinoma after the endoscopic sclerotherapy of varices [abstract] Span J Dig Dis 1992 8 43 46 1520550

[B6] Ohta M Kuwano H Hashizume M Sonoda T Tomikawa M Higashi H Ohno S Watanabe M Sugimachi K Development of esophageal cancer after endoscopic injection sclerotherapy for esophageal varices: three case reports Endoscopy 1995 8 455 458 10.1055/s-2007-1005742 8549446

[B7] Tanoue K Hashizume M Ohta M Ueno K Kitano S Sugimachi K Development of early squamous cell carcinoma of the esophagus after endoscopic injection sclerotherapy for esophageal varices [abstract] Hepatogastroenterology 1995 8 792 796 8847025

[B8] Ng KW Tan SW Chen YH Chen HC Wu CS Liang CT Jiang CF Esophageal cancer after endoscopic injection sclerotherapy for esophageal varices [abstract] Zhonghua Yi Xue Za Zhi (Taipei) 2001 8 299 304 11499340

[B9] Hashimoto Y Imai A Tokui N Sasaki A Saitoh H Koie T Ohyama C Unexpected outcome after sclerotherapy of simple renal cyst BMC Neurol 2012 8 63 10.1186/1471-2202-13-63 PMC3467159 22827879

[B10] Baek HJ Hong JP Choi JW Suh DC Direct percutaneous alcohol sclerotherapy for venous malformations of head and neck region without fluoroscopic guidance: technical consideration and outcome Neurointervention 2011 8 84 88 10.5469/neuroint.2011.6.2.84 22125754 PMC3214818

[B11] Zheng JW Yang XJ Wang YA He Y Ye WM Zhang ZY Intralesional injection of pingyangmycin for vascular malformations in oral and maxillofacial regions: an evaluation of 297 consecutive patients Oral oncology 2009 8 872 876 10.1016/j.oraloncology.2009.02.011 19628423

[B12] Zhao JH Zhang WF Zhao YF Sclerotherapy of oral and facial venous malformations with use of pingyangmycin and/or sodium morrhuate Int J Oral Maxillofac Surg 2004 8 463 466 10.1016/j.ijom.2003.10.003 15183410

[B13] Hemmings C Is carcinoma a mesenchymal disease? The role of the stromal microenvironment in carcinogenesis Pathology 2013 8 371 381 10.1097/PAT.0b013e328360b600 23594691

[B14] Hashimoto N Phan SH Imaizumi K Matsuo M Nakashima H Kawabe T Shimokata K Hasegawa Y Endothelial-mesenchymal transition in bleomycin-induced pulmonary fibrosis Am J Respir Cell Mol Biol 2010 8 161 172 10.1165/rcmb.2009-0031OC 19767450 PMC2937229

[B15] Zhang W Chen G Ren JG Zhao YF Bleomycin induces endothelial mesenchymal transition through activation of mTOR pathway: a possible mechanism contributing to the sclerotherapy of venous malformations Br J Pharmacol 2013 doi:10.1111/bph.12355 [Epub ahead of print] 10.1111/bph.12355 PMC3838696 23992520

[B16] Liu Y Dong XL Tian C Liu HG Human telomerase RNA component (hTERC) gene amplification detected by FISH in precancerous lesions and carcinoma of the larynx Diagn Pathol 2012 8 34 10.1186/1746-1596-7-34 22463766 PMC3359179

[B17] Li G Jin T Liang H Tu Y Zhang W Gong L Su Q Gao G Skewed X-chromosome inactivation in patients with esophageal carcinoma Diagn Pathol 2012 8 55 23556484 10.1186/1746-1596-8-55 PMC3640911

[B18] El Fatemi H Chahbouni S Jayi S Moumna K Melhouf MA Bannani A Mesbahi O Amarti A Luminal B tumors are the most frequent molecular subtype in breast cancer of North African women: an immunohistochemical profile study from Morocco Diagn Pathol 2012 8 170 10.1186/1746-1596-7-170 23216981 PMC3538531

